# Retinitis Pigmentosa and Therapeutic Approaches: A Systematic Review

**DOI:** 10.3390/jcm13164680

**Published:** 2024-08-09

**Authors:** Filippo Confalonieri, Antonio La Rosa, Giovanni Ottonelli, Gianmaria Barone, Vanessa Ferraro, Alessandra Di Maria, Mary Romano, Alessandro Randazzo, Josè Luis Vallejo-Garcia, Paolo Vinciguerra, Goran Petrovski

**Affiliations:** 1 Department of Biomedical Sciences, Humanitas University, Pieve Emanuele, 20090 Milan, Italy; 2 Department of Ophthalmology, IRCCS Humanitas Research Hospital, Rozzano, 20089 Milan, Italy; 3 Center for Eye Research and Innovative Diagnostics, Department of Ophthalmology, Institute for Clinical Medicine, University of Oslo, Kirkeveien 166, 0450 Oslo, Norway; 4 Department of Ophthalmology, Oslo University Hospital, Kirkeveien 166, 0450 Oslo, Norway; 5 Department of Ophthalmology, University of Split School of Medicine and University Hospital Centre, 21000 Split, Croatia; 6 UKLONetwork, University St. Kliment Ohridski-Bitola, 7000 Bitola, North Macedonia

**Keywords:** retinitis pigmentosa, retinal disease, gene therapy, inherited retinal dystrophy

## Abstract

**Background:** Retinitis pigmentosa (RP) is a group of hereditary retinal dystrophies characterized by progressive degeneration of photoreceptor cells, which results in debilitating visual impairment. This systematic review aims to evaluate the efficacy and safety of emerging treatment modalities for RP, including gene therapy, mesenchymal-cell-based approaches, and supplementary interventions. **Methods:** A comprehensive search of electronic databases was conducted to identify relevant studies published up to February 2024. Studies reporting outcomes of treatment interventions for RP, including randomized controlled trials, non-randomized studies, and case series, were included. Data extraction and synthesis were performed according to predefined criteria, focusing on assessing the quality of evidence and summarizing key findings. **Results:** The search yielded 13 studies meeting inclusion criteria, encompassing diverse treatment modalities and study designs. Gene therapy emerged as a promising therapeutic approach, with several studies reporting favorable outcomes regarding visual function preservation and disease stabilization. Mesenchymal-cell-based therapies also demonstrated potential benefits, although evidence remains limited and heterogeneous. Supplementary interventions, including nutritional supplements and neuroprotective agents, exhibited variable efficacy, with conflicting findings across studies. **Conclusions:** Despite the lack of definitive curative treatments, emerging therapeutic modalities promise to slow disease progression and preserve visual function in individuals with RP. However, substantial gaps in evidence and heterogeneity in study methodologies underscore the need for further research to elucidate optimal treatment strategies, refine patient selection criteria, and enhance long-term outcomes. This systematic review provides a comprehensive synthesis of current evidence and highlights directions for future research to advance the care and management of individuals with RP.

## 1. Introduction

Retinitis pigmentosa (RP) is one of the most prevalent inherited retinal dystrophies worldwide, encompassing a group of genetically heterogeneous disorders primarily affecting the photoreceptor cells within the retina [1]. It is estimated to affect more than 1.5 million patients worldwide, with varying prevalence across different populations and ethnicities [2]. This condition is characterized by progressive degeneration of the photoreceptor cells, leading to debilitating visual impairment and often culminating in legal blindness in its advanced stages. The hallmark clinical features of RP typically manifest as night blindness, followed by gradual constriction of the visual field, decreased visual acuity, and, in some cases, the eventual loss of central vision. Electroretinography (ERG) often reveals reduced rod photoreceptor responses with reduced, to a lesser extent, cone photoreceptor responses, further corroborating the diagnosis. While RP can be present in isolation, it can also occur as part of syndromic disorders, such as Usher syndrome or Bardet–Biedl syndrome, or as a component of complex genetic syndromes.

The prognosis for individuals affected by RP varies widely based on genetic subtype, age of onset, and the rate of disease progression [1,2]. Despite decades of research and considerable advancements in understanding the molecular mechanisms underpinning RP, efficacious treatment options still need to be discovered. Traditional management strategies have primarily focused on supportive measures to preserve existing vision and enhance visual function through low-vision aids and adaptive techniques. However, these interventions do not alter the natural course of the disease nor halt its progression [3]. Given the significant burden imposed by RP on affected individuals and their families, there is an urgent need to explore novel therapeutic avenues that address the underlying pathophysiology of the disease. Recent years have witnessed increasing interest and substantial investment in developing gene-based therapies, cellular transplantation strategies, and supplementation regimens targeting retinal degenerative processes. These emerging therapeutic modalities hold promise in potentially slowing or halting disease progression, thereby offering hope for preserving vision and improving the quality of life for individuals living with RP [4].

In this systematic review, we aim to comprehensively evaluate the current landscape of treatment modalities for RP, with a particular focus on gene therapy, mesenchymal-cell-based approaches, and supplementary interventions. Through a meticulous synthesis of existing literature and critical appraisal of clinical evidence, we endeavor to provide insights into these therapeutic interventions’ efficacy, safety, and prospects in managing RP. Such an appraisal is essential for informing clinical decision making, guiding research priorities, and ultimately advancing the care and outcomes for individuals affected with this debilitating retinal disorder.

## 2. Materials and Methods

A systematic review was performed to determine the current therapeutic approaches evaluated by trials in treating patients with retinitis pigmentosa.

The review followed the guidelines established by the Preferred Reporting Items for Systematic Reviews and Meta-Analyses (PRISMA), which provide a framework for transparent and comprehensive reporting of systematic reviews.

To identify relevant articles, we conducted a systematic literature search from the inception in 1946 to 20 February 2024 using a controlled vocabulary and specific keywords related to “retinitis pigmentosa”, “mesenchymal”, “mesenchymal stem cells”, “stem cell transplantation”, “intravitreal”, “intravitreal therapy”, “gene therapy”, “gene vectors”, “surgical approaches”, and “clinical trials”. The search was conducted in electronic databases such as Ovid Medline, Embase (Ovid), the Cochrane Register of Controlled Trials, and the Cochrane Database of Systematic Reviews. The “Rayyan” software (version 2022, Cambridge, MA, USA, 2022) was used as an automation tool to select articles [5].

This process allowed us to ensure complete coverage of all available literature. The complete search strategy is given in Appendix A.

After compiling the electronic article list, two reviewers (O.G. and A.L.R.) independently evaluated all the abstracts one by one and identified the articles that met the inclusion criteria. These criteria included all the studies that evaluated the efficacy of any therapeutic approaches and clinical outcomes for patients with retinitis pigmentosa.

Exclusion criteria were review studies, pilot studies, case series, case reports, photo essays, expert opinions, and studies written in languages other than English. Studies conducted on animal eyes and cadavers were also excluded. No unpublished data were requested from the corresponding authors of the selected articles; the analysis was conducted solely based on available published information. A consensus was reached in case of disagreement among reviewers, and an expert reviewer (F.C.) was consulted if necessary to provide additional expertise. The determining reasons for inclusion or exclusion of the full-text reviewed articles are summarized in Appendix B.

The 2011 Oxford Centre for Evidence-Based Medicine (OCEBM) guidelines [6] were followed to assess the strength of evidence. These recommendations provide a methodological framework to evaluate the reliability and validity of evidence in medical research. The Grading of Recommendations Assessment, Development, and Evaluation (GRADE) system [7], a tool created to assess the integrity of evidence and facilitate the formulation of recommendations, was used to determine the quality of evidence.

In summary, our systematic review strictly followed the methodologies outlined in the PRISMA guidelines. It adopted a comprehensive search strategy to identify relevant studies, clearly delineated inclusion and exclusion criteria, and used established criteria to assess the level and quality of evidence.

**Figure 1:**
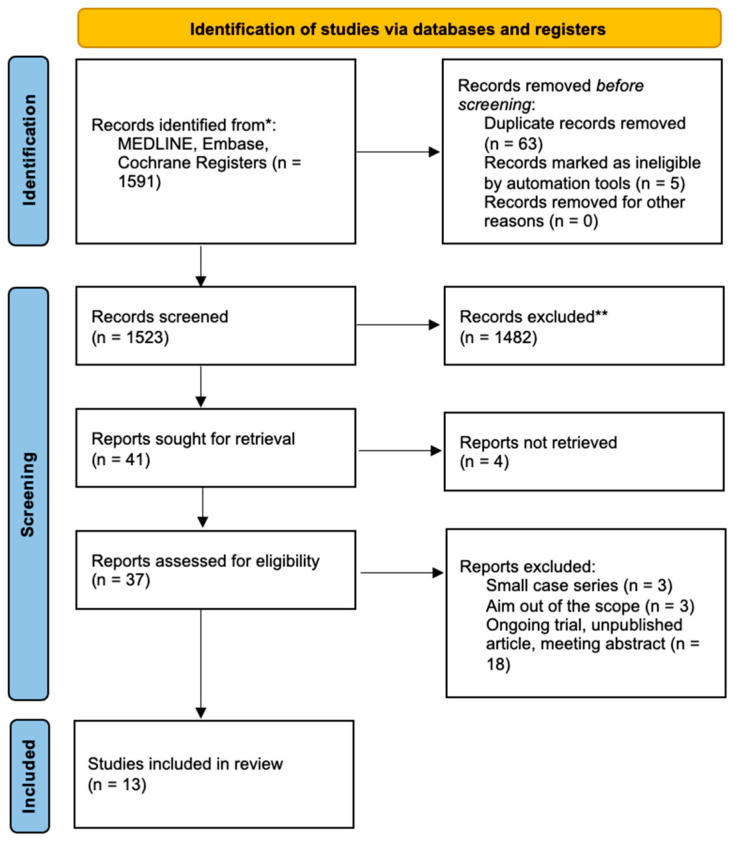
Flowchart of the literature search and selection according to Preferred Reporting Items for Systematic Reviews and Meta-Analyses (PRISMA). * Consider, if feasible to do so, reporting the number of records identified from each database or register searched (rather than the total number across all databases/registers). ** If automation tools were used, indicate how many records were excluded by a human and how many were excluded by automation tools.

Figure 1 summarizes the research approach applied in this systematic review within a flowchart.

## 3. Results

### 3.1. Study Characteristics

Across the thirteen extracted studies [8,9,10,11,12,13,14,15,16,17,18,19,20], 917 eyes were treated, providing a substantial dataset for analysis. The average age of participants ranged between 40 and 50 years across different trials, with specific studies targeting adult populations and others encompassing a broader age range [16,17].

Gender distribution was equally represented across the studies, ensuring a balanced sample. On average, approximately 50% of participants were male, and 50% were female, reflecting the inclusivity of the research. Regarding the severity of retinitis pigmentosa (RP) among participants, most studies included individuals with advanced RP or RP complicated by macular edema. However, some trials incorporated patients with varying degrees of disease severity, from early-stage to advanced RP [8,9].

Various treatment modalities were explored regarding the therapeutic method of investigating retinitis pigmentosa (RP). Mesenchymal stem cell (MSC) therapy emerged prominently, with six studies investigating its potential in preserving or improving visual function [8,9,10,11,12,13]. Gene therapies also garnered attention, focusing on correcting genetic mutations associated with RP in two studies [14,15]. Additionally, dietary supplementation with docosahexaenoic acid (DHA) was examined in two trials [16,17]. Pharmacological interventions, including oral valproic acid (VPA) and oral QLT091001, were explored in separate studies [18,19]. Finally, one trial investigated visual implants as a novel treatment modality [20].

A comprehensive data synthesis was unavailable due to the diversity of the available data and differences in research designs.

Studies included are hereby subdivided in the following sections and summarized in Table 1.

### 3.2. Mesenchymal Stem Cells

In a study by Zhao et al. (2020) [8], 32 adult patients (64 eyes) underwent intravenous infusion of human umbilical cord MSCs (UCMSCs) with a follow-up period of 12 months. The treatment involved a single injection of 10^8^ UCMSCs, and evaluations included safety assessments, central macular thickness (CMT), visual field sensitivity, best-corrected visual acuity (BCVA), flash visual evoked potential (FVEP), and the NEI-VFQ-25 questionnaire. The results indicated a good tolerance of UCMSC infusion, with no adverse effects observed. Although there were non-significant changes in macular thickness and BCVA, the NEI-VFQ-25 scores significantly improved three months posttreatment (*p* < 0.05), suggesting short-term therapeutic effects mainly within the initial three months.

In another study by Zhao et al. (2021) [9], 20 adult patients (40 eyes) with RP complicated by macular edema were enrolled to compare the efficacy of intravenous UCMSC infusion with modified sub-Tenon’s capsule injection of triamcinolone acetonide (TA). The follow-up duration was six months. Both treatments demonstrated safety, with no severe adverse effects observed. While TA rapidly reduced CMT in the short term, UCMSC infusion offered longer-lasting benefits, with a more significant reduction in CMT observed at six months (*p* < 0.05). Additionally, UCMSCs showed a significantly greater FVEP amplitude growth rate than TA at six months (*p* < 0.05), indicating superior therapeutic potential in improving visual function.

Kahraman et al. (2020) [10] conducted a study involving 82 RP patients (124 eyes) who underwent a surgical procedure to inject 5 million UCMSCs into the suprachoroidal area. This intervention resulted in statistically significant improvements in BCVA and visual field (VF) tests over a 6-month follow-up period (*p* < 0.05). Additionally, multifocal electroretinography (mfERG) recordings showed significant improvements in the amplitudes of P1 waves in the central areas, indicating positive effects on retinal function.

Ozmert et al. (2023) [11] investigated the effects of sub-Tenon WJ-MSC injection, Magnovision stimulation, and their combination in 80 RP patients (130 eyes). The study found that FAF-field delta changes were 0.39 mm^2^ in the WJ-MSC-only group, 1.50 mm^2^ in the Magnovision-only group, 0.07 mm^2^ in the combined management group, and 12.04 mm^2^ in the control group (*p* < 0.05).

Tuekprakhon et al. (2021) [12] conducted a prospective, open-label, non-randomized phase I clinical trial involving 14 adult RP patients who underwent intravitreal injection of autologous BM-MSCs aspirated from the patient posterior iliac crest. The participants were divided into three intervention groups based on the quantity of MSCs injected: Group 1 received 1 × 10^6^ cells, Group 2 received 5 × 10^6^ cells, and Group 3 received 1 × 10^7^ cells. Group 1 showed the most significant improvement compared to the fellow eye, with statistically significant improvements observed at months 7 and 8. Group 3 displayed immediate significant BCVA improvement at month 1, returning to baseline by month 6. Additionally, most participants reported subjective improvements in quality of life.

A study by Limoli et al. (2020) [13] involved 25 RP patients who underwent an autograft of mesenchymal cells, fat cells, and platelet-rich plasma (PRP) using LRRT. The patients were subdivided based on central retinal thickness, and improvements in BCVA were observed in both groups, with statistically significant improvements noted in Group A (*p* < 0.05).

### 3.3. Gene Therapy

Kapetanovic et al. (2020) [14] enrolled 18 patients with X-linked retinitis pigmentosa (RP) caused by mutations in RPGR. The treatment involved the delivery of increasing concentrations of a codon-optimized AAV2 serotype 8 vectors (AAV8.coRPGR) into the subretinal space via a two-step injection. The primary safety endpoint was the incidence of dose-limiting toxicities and treatment-emergent adverse events over 24 months, with secondary endpoints including changes in retinal sensitivity, best-corrected visual acuity (BCVA), SD-OCT, and autofluorescence over the same period. The study revealed a dose–response effect across the trial cohorts regarding gains in visual function in treated eyes. Patients who received mid-dose injections of AAV8.coRPGR showed gains in retinal sensitivity and reversal of some visual field loss by month 1, with sustained treatment effects observed through to the 6-month follow-up. All patients reported subjective improvements in visual clarity and an increase in the field of vision within one month of follow-up. However, early cohort patients with advanced retinal degeneration showed no noticeable gains in visual function at low vector doses, although visual acuity returned to baseline levels by three months postsurgery.

In the study by Albert M. Maguire et al. (2021) [15], which involved 29 patients with RPE65 mutation-associated inherited retinal dystrophy (IRD), gene augmentation therapy was administered using the recombinant adeno-associated viral vector Voretigene Neparvovec. Patients were randomized into original intervention and delayed intervention control groups, with assessments conducted at various intervals over five years. The intervention group received 1.5 × 10^11^ vg of VN in each eye, while the control group underwent delayed intervention after one year. The results showed consistent but insignificant improvements in ambulatory navigation, light sensitivity, and visual field over 3 to 4 years compared to baseline in both groups. No significant differences were observed between the two groups regarding efficacy. Safety assessments revealed no product-related serious adverse events, although one delayed intervention group patient experienced loss of foveal function attributed to the administration procedure.

### 3.4. Docoexhanoic Acid

The DHAX trial by Dennis R. Hoffman et al. (2015) [16], a single-site, placebo-controlled, randomized clinical trial involving 51 patients with X-linked retinitis pigmentosa (XLRP), comprised 29 treated and 22 placebo groups. The trial investigated the efficacy of oral docosahexaenoic acid (DHA) supplementation over four years. The treatment group received 30 mg DHA/kg/d, while the placebo group received a placebo. Visual outcomes were measured annually, and red blood cell (RBC) DHA levels were determined every six months. Oral DHA supplementation significantly increased mean RBC-DHA levels by 4-fold over placebo (*p* < 0.0001). While no significant group differences were found for visual acuity, shape discrimination, or fundus appearance, DHA supplementation showed substantial reductions in the yearly progression rates for dark-adapted thresholds and visual field sensitivity in various regions (*p* = 0.06 to <0.0001). Visual field sensitivity decline rates depended on RBC-DHA levels (*p* = 0.046 to <0.0001).

Similarly, a previous trial by Dennis R. Hoffman et al. (2003) [17] was a 4-year prospective randomized clinical trial that enrolled male patients with XLRP (mean age = 16 years; range = 4–38 years) who received either DHA (400 mg/d) or placebo. The study assessed red blood cell (RBC)-DHA concentrations every six months and recorded full-field cone electroretinograms (ERGs), visual acuity, dark adaptation, visual fields, rod ERGs, and fundus photos annually. The +DHA group exhibited a 2.5-fold increase in RBC-DHA levels over placebo (70 vs. 28 mg DHA/L). While there was no significant difference in the rate of cone ERG functional loss between groups, DHA supplementation was beneficial in reducing rod ERG functional loss in patients aged < 12 years (*p* = 0.040) and preserving cone ERG function in patients ≥ 12 years (*p* = 0.038).

### 3.5. Oral Valproic Acid

Birch et al. (2018) [18] conducted a multicenter, phase II, prospective, interventional, placebo-controlled, double-masked randomized clinical trial involving 90 male and female patients with autosomal dominant retinitis pigmentosa (AD-RP). Participants were randomized to receive either oral valproic acid (VPA) at doses ranging from 500 mg to 1000 mg daily (*n* = 46) or placebo (*n* = 44) for 12 months. Follow-up visits were scheduled at 8, 26, 39, 52, and 65 weeks. The primary outcome measure was the change in kinetic perimetry (KP) visual field area (VFA) between baseline and week 52, assessed by the III4e isopter, with greater sensitivity indicating improvement.

The study did not meet its primary endpoint, as there was no significant change in visual field area between the VPA and placebo groups at 12 months. Furthermore, the two groups observed no substantial improvement in the secondary outcome measures. Consequently, the study concluded no support for using valproic acid to improve visual function in patients with AD-RP.

### 3.6. Oral QLT091001

Scholl et al. (2015) [19] investigated the effects of oral QLT091001 in 18 patients with retinitis pigmentosa (RP) resulting from mutations in the RPE65 or LRAT genes, aged 5 to 65 years. After a 7-day course of QLT091001, ocular examinations revealed promising outcomes. Within two months of treatment, 44% of participants experienced a 20% increase in the functional retinal area, with 22% showing a 40% increase. Additionally, 67% of patients demonstrated a 5-letter increase in visual acuity, with 28% experiencing a 10-letter increase. A significant difference was observed in the length of the outer segment layer between treatment responders (average of 11.7 μm) and non-responders (average of 3.5 μm), indicating a positive treatment effect (*p* = 0.02). These findings highlight the potential efficacy of oral QLT091001 in improving visual function in RP patients with RPE65 or LRAT mutations, alongside its acceptable safety profile.

### 3.7. Retina Implant Alpha IMS

Stingl et al. (2017) [20] enrolled 29 patients grappling with hereditary retinal degeneration who received the Retina Implant Alpha IMS. Over a 1-year follow-up period, noteworthy outcomes were observed: 72% of participants exhibited enhancements in their daily activities and mobility, indicating a substantial improvement in their quality of life. Moreover, as much as 86% reported improved visual acuity and object recognition, showcasing the transformative potential of the implant (*p* < 0.05). Participants demonstrated significant improvements in detecting, localizing, and recognizing shapes and objects with the implant activated (*p* < 0.05). Despite initial challenges, 86% showed remarkable progress in light perception with the implant, underscoring its efficacy in restoring this critical visual function (*p* < 0.05). Management of adverse events ensured the safety and well-being of all participants throughout the study period, and no significant adverse events were reported.

## 4. Discussion

In RP, where clinical trials have explored various therapeutic strategies, our systematic review tried to consolidate all significant trials, providing valuable insights into their potential efficacy and challenges [21,22,23,24].

Gene therapy, exemplified by voretigene neparvovec (Luxturna), resulted in the most promising avenue for improving visual function and slowing RP progression [14,15]. Similarly, CRISPR/Cas9 gene editing and RNA interference hold promise for targeted treatment strategies tailored to specific genetic mutations associated with RP in humans, even though only animal-model-based studies hold validated scientific results for now [25,26,27,28].

Cell-based therapies, particularly mesenchymal stem cell (MSC) transplantation, have demonstrated safety and efficacy in preserving retinal structure and function in preclinical models and early-phase clinical trials [29,30]. The neuroprotective and immunomodulatory properties of MSCs offer additional benefits in mitigating retinal degeneration [31,32,33,34,35,36,37].

Neuroprotective agents, such as valproic acid (VPA), have shown potential in preclinical studies by modulating neuroinflammation and promoting neuronal survival [38]. However, clinical trials evaluating VPA in RP patients have yielded conflicting results, necessitating further investigation [18].

Emerging therapeutic targets, including small-molecule drugs that target oxidative stress, apoptosis, and inflammation, are paving the way for future RP treatments [39,40,41,42,43]. Additionally, optogenetic therapies that utilize light-sensitive proteins offer hope for enhancing visual function in RP patients [44,45,46,47].

Another possibility to treat RP patients is the implantation of a retinal prosthesis [48,49,50]. Despite the promising advancements in retinal prostheses, each device comes with specific benefits and drawbacks [51]. The long-term effects, safety, and durability of these devices remain uncertain. Additionally, financial constraints and a shift in focus towards visual cortical implants have led to the discontinuation of production by leading companies such as ARGUS II. This shift is primarily due to limited resources and the relatively small patient population that qualifies for retinal prostheses compared to visual cortical implants [52].

The pool of eligible candidates is further narrowed by the need for patients to be both physically and psychologically fit for surgery and postimplant rehabilitation [53]. The vision provided by retinal prostheses is notably different from natural vision, requiring patients to have a strong support system, a comprehensive understanding of expected outcomes, and additional training and practice to optimize results. Furthermore, patients often need to travel significant distances for surgery, frequently out of state, and stay away from home for extended periods during the pre- and postsurgical phases [54].

As more research is conducted, the impact of these devices once they are commercially available remains uncertain. Social determinants of health (SDOHs) may hinder low-resource patients with retinitis pigmentosa (RP) from accessing these technologies. Although there are a few studies indicating a higher prevalence of RP in specific marginalized groups, cost and availability pose significant barriers for patients needing this equipment. Numerous challenges must be addressed before these devices can become commercially viable, including a thorough analysis of financial implications to ensure equitable access to care. Moreover, there is limited research on the long-term maintenance costs of these devices, and some have been discontinued while still implanted in patients [55].

The promising outcomes from studies on retinal prostheses have spurred interest in their potential applications for other vision-threatening conditions. The ORION II device by Vivani Medical, Inc. is currently being evaluated for its effectiveness in restoring vision in patients with glaucoma, diabetic retinopathy, optic nerve injuries, cancer, and trauma. Similarly, the PRIMA device and IMTC’s HARP4k Retinal Prosthesis System are being studied for their potential in treating diseases that cause photoreceptor degeneration, particularly advanced atrophic dry age-related macular degeneration [56]. The NR600 and BVA devices have shown promise for patients with RP and age-related macular degeneration, while the ICVP system is being investigated for its use in cases of ocular injuries, optic nerve diseases, photoreceptor degeneration, and blindness [57].

Despite these advancements, translating experimental treatments into clinical practice remains challenging. Long-term safety and efficacy data, optimized delivery methods, and consideration of RP’s genetic heterogeneity are needed [58]. Furthermore, the high cost of gene- and cell-based therapies presents economic barriers to widespread adoption.

## 5. Conclusions

In conclusion, while significant progress has been made in understanding and treating RP, further research is needed to elucidate optimal treatment modalities and ensure patient accessibility. By addressing these challenges through interdisciplinary collaboration and technological advances, effective treatments for RP may become a reality, offering hope to those affected by this debilitating disease.

## Figures and Tables

**Table 1:** Summary of Included Studies and Salient Features.

*N*°	Title	Author (Year)	Study Design	Study Sample	Investigated Treatment	Methods	Outcomes	Main Findings	GRADE
1	Intravenous Infusion of Umbilical Cord Mesenchymal Stem Cells Maintains and Partially Improves Visual Function in Patients with Advanced Retinitis Pigmentosa	Zhao T. et al. (2020) [8]	Prospective, open label, single-arm, phase I/II clinical trial	32 adult patients (64 eyes), male and female	Intravenous infusion of human umbilical cord mesenchymal stem cells (UCMSCs)	Single infusion of 10^8^ UCMSCs (250 mL); 12-month follow-up; evaluated safety, CMT, visual field sensitivity, BCVA, FVEP, and NEI-VFQ-25 scores at set intervals	No adverse effects Stable CMT Non-significant BCVA increase No significant change in visual field sensitivity and FVEP Improved NEI-VFQ-25 at 3 months	UCMSC infusion well-tolerated; short-term improvement in BCVA and quality of life; significant NEI-VFQ-25 improvement at 3 months; no significant long-term effects on visual field sensitivity or FVEP. Short-term benefits likely due to diminishing functional properties over time	Moderate
2	Comparative Study of a Modified Sub-Tenon’s Capsule Injection of Triamcinolone Acetonide and the Intravenous Infusion of Umbilical Cord Mesenchymal Stem Cells in Retinitis Pigmentosa Combined With Macular Edema	Zhao T. et al. (2021) [9]	Prospective, open label, randomized, phase I/II clinical trial	20 adult patients (40 eyes), male and female	Comparison of sub-Tenon’s injection of triamcinolone acetonide (TA) and intravenous infusion of umbilical cord mesenchymal stem cells (UCMSCs) in RP patients with macular edema	TA: 20 mg injection; UCMSCs: 3 × 10^6^ (250 mL) infusion; 6-month follow-up; evaluated safety, CMT, visual field sensitivity, BCVA, and FVEP	No severe adverse effects in both groups TA reduced CMT significantly at 1 week, 1 month, and 2 months; UCMSCs at 1 month and had greater reduction at 6 months TA increased FVEP P2 at 2 months; UCMSCs at 6 months No significant visual acuity or field differences	Both treatments were safe. TA reduced macular edema quickly; UCMSCs had longer-lasting effects and better visual function improvement over time	Moderate
3	Umbilical cord derived mesenchymal stem cell implantation in retinitis pigmentosa: a 6-month follow-up results of a phase 3 trial	Neslihan Sinim Kahraman (2020) [10]	Prospective, single-center, phase III clinical study	82 patients (124 eyes)	5 million UCMSCs injected into the suprachoroidal area via surgery	Injection by experienced surgeon; evaluations at presurgery, 1 day, 1 week, 1-, 3-, and 6-months postsurgery	No serious systemic or ocular complications Significant improvements in BCVA and VF (*p* < 0.05) Significant improvements in mfERG P1 wave amplitudes and implicit times in central areas	Safe procedure with significant improvements in visual acuity, visual field, and retinal function	Moderate
4	Management of Retinitis Pigmentosa Via Wharton’s Jelly-Derived Mesenchymal Stem Cells or Combination With Magnovision: 3-Year Prospective Results	Ozmert (2023) [11]	Prospective, sequential, open-label clinical study	80 patients (130 eyes)	Comparison of sub-Tenon WJ-MSC-only, Magnovision-only, combined WJ-MSC and Magnovision, and control groups in RP patients	Group 1: Sub-Tenon WJ-MSC (34 eyes)—Group 2: Magnovision (32 eyes)—Group 3: Combined WJ-MSC and Magnovision (32 eyes)—Group 4: Control (no treatment, 32 eyes)	Primary: Fundus autofluorescence surface area (FAF-field) Secondary: ETDRS visual acuity (BCVA), ellipsoid zone widths (EZWs), fundus perimetry deviation index (FPDI), full-field multiluminance ERG	FAF-field changes: Group 1: 0.39 mm^2^, Group 2: 1.50 mm^2^, Group 3: 0.07 mm^2^, Group 4: 12.04 mm^2^ EZW, BCVA, FPDI changes: Group 4 > Groups 1, 2 > Group 3—ERG-m changes: Group 3 > Groups 1, 2, 4	High
5	Intravitreal autologous mesenchymal stem cell transplantation: a non-randomized phase I clinical trial in patients with retinitis pigmentosa	Tuekprakhon et al. (2021) [12]	Prospective, open-label, non-randomized phase I clinical trial	14 adult patients (14 eyes treated, fellow eye control), male and female	Intravitreal injection of autologous BM-MSCs from posterior iliac crest	Single injection in right eye; left eye as control; 3 groups based on MSC quantity (1 × 10^6^ cells, 5 × 10^6^ cells, and 1 × 10^7^ cells)	Primary: Safety assessed through various measures Secondary: BCVA, visual fields, central subfield thickness, ERG	No serious adverse events Stable IOP Transient increase in anterior chamber cells and flare Slight BCVA improvement Subjective quality of life improvements reported	Low
6	Mesenchymal stem cell surgery, rescue and regeneration in retinitis pigmentosa: clinical and rehabilitative prognostic aspects	Limoli et al. (2020) [13]	Retrospective clinical study	25 patients, 11 women and 14 men, with an average age of 45.9 ± 18.36 years (34 eyes)	Autograft of mesenchymal cells of fat cells and PRP using LRRT (between the choroid and sclera)	All eyes were divided into two groups based on central retinal thickness (FT recorded by SD-OCT): Group A (≤190 μm) and Group B (>190 μm)	Mean BCVA, mean close-up visus, average threshold sensitivity, average threshold of close-up visus with magnifying system, percentage of change	BCVA changes: Group A varied from 0.89 to 0.85 logMAR (+4.16%, *p* = 0.9701); Group B varied from 0.45 to 0.37 logMAR (+16.31%, *p* = 0.9083)	Moderate
7	Initial results from a first-in-human gene therapy trial on X-linked retinitis pigmentosa caused by mutations in RPGR	Kapetanovic (2020) [14]	Gene therapy trial	18 patients	Increasing concentrations of codon-optimized AAV2 serotype 8 vector (AAV8.coRPGR)	Vector delivery into subretinal space via two-step injection	Primary safety endpoint: Incidence of dose-limiting toxicities and treatment-emergent adverse events over 24 months. Secondary endpoints: Changes in retinal sensitivity, BCVA, SD-OCT, and autofluorescence over 24 months	Dose–response effects observed, with mid-dose patients showing gains in retinal sensitivity and visual field improvement. Visual acuity returned to baseline levels by 3 months postsurgery. Subjective improvement in visual clarity and field of vision reported by all patients at 1-month follow-up. Functional assessment showed similar visual acuity to baseline	Moderate
8	Durability of Voretigene Neparvovec for Biallelic RPE65-Mediated Inherited Retinal Disease Phase 3 Results at 3 and 4 Years	Albert M Maguire et al. (2021) [15]	Open-label, randomized, controlled phase III trial	29 male and female patients with RPE65-mutated IRD	Gene augmentation therapy with recombinant AAV vector voretigene neparvovec-rzyl (VN)	Randomization between original intervention (*n* = 19) and delayed intervention control (*n* = 10). Treatment: Intervention group received 1.5 × 10^11^ vg of VN in each eye. Controls switched to intervention after 1 year	Long-term efficacy and safety assessment over 5 years: multiluminance mobility test, full-field light sensitivity threshold, visual field, and visual acuity	Safety: No product-related serious adverse events Both groups showed consistent but not significant improvements in ambulatory navigation, light sensitivity, and visual field over 3 to 4 years compared to baseline One delayed intervention group patient experienced foveal loss attributed to the administration procedure	High
9	Docosahexaenoic Acid Slows Visual Field Progression in X-Linked Retinitis Pigmentosa: Ancillary Outcomes of the DHAX Trial	Dennis R. Hoffman et al. (2015) [16]	Single-site, placebo-controlled, randomized clinical trial	51 patients (29 treated and 22 placebo)	Oral DHA supplementation	XLRP patients (age: 7–31) received 30 mg/kg/d or placebo for 4 years. Follow-up: Visual outcomes annually; RBC-DHA every 6 months	RBC-DHA levels increased 4-fold over placebo (*p* < 0.0001) No significant differences in visual acuity, shape discrimination, or fundus appearance Reduced progression in dark-adapted thresholds and visual field sensitivity with DHA supplementation (*p* < 0.05)	No significant changes in ERG function between groups DHA supplementation showed less progression in dark-adapted thresholds compared to placebo over 4 years Significant reductions in disease progression rates for various visual field measures with DHA supplementation	High
10	A Randomized, Placebo-controlled Clinical Trial of Docosahexaenoic Acid Supplementation for X-linked Retinitis Pigmentosa	Dennis R. Hoffman et al. (2003) [17]	4-year prospective randomized clinical trial	44 male patients with XLRP (mean age = 16 years; range = 4–38 years) received DHA (400 mg/d; *n* = 23; +DHA group) or placebo (*n* = 21)	Oral supplementation of docosahexaenoic acid	Male patients with XLRP (mean age = 16 years; range = 4–38 years) received DHA (400 mg/d; *n* = 23; +DHA group) or placebo (*n* = 21). Follow-up: RBC-DHA concentrations assessed every 6 months. Full-field cone ERGs, visual acuity, dark adaptation, visual fields, rod ERGs, and fundus photos recorded annually	RBC-DHA increased 2.5-fold in +DHA group No significant difference in cone ERG function between groups Preservation of cone ERG function correlated with RBC-DHA Less change in fundus appearance in +DHA group Subset analysis showed DHA supplementation reduced rod ERG loss in patients aged < 12 years and preserved cone ERG function in patients ≥ 12 years	Although DHA-supplemented patients had significantly higher RBC-DHA levels, cone ERG functional loss rate was not significantly different between groups	High
11	Effect of Oral Valproic Acid vs. Placebo for Vision Loss in Patients With Autosomal Dominant Retinitis Pigmentosa A Randomized Phase 2 Multicenter Placebo-Controlled Clinical Trial	David G. Birch et al. (2018) [18]	Multicenter, phase II, prospective, interventional, placebo-controlled, double-masked randomized clinical trial	90 male and female patients with autosomal dominant RP	Oral VPA 500–1000 mg daily	Participants randomized to receive VPA (*n*= 46) or placebo (*n* = 44) for 12 months. Dose selection based on proof-of-concept studies. Follow-up visits at 8, 26, 39, 52, and 65 weeks	Primary outcome: Change in kinetic perimetry (KP) visual field area (VFA) assessed by the III4e isopter between baseline and week 52. Secondary outcomes: Visual function measures	The study did not meet its primary endpoint at 12 months, showing no change in visual field area between groups No significant improvement in any secondary outcomes observed between the two groups The study does not support the use of valproic acid to enhance visual function in AD-RP patients	Very high
12	Safety and Proof-of-Concept Study of Oral QLT091001 in Retinitis Pigmentosa Due to Inherited Deficiencies of Retinal Pigment Epithelial 65 Protein (RPE65) or Lecithin: Retinol Acyltransferase (LRAT)	Hendrik P. *n*. Scholl et al. (2015) [19]	International, multicenter, open-label, proof-of-concept study	18 patients with RPE65- or LRAT-related retinitis pigmentosa with autosomal recessive RP due to biallelic mutations in either the RPE65 or LRAT gene confirmed in an accredited molecular genetic laboratory and between 5 and 65 years of age	Oral QLT091001	Patients received 40 mg/m^2^/day QLT091001 for 7 days	Within 2 months, 44% showed a 20% increase in retinal area 67% showed a 5-letter ETDRS score increase	Baseline outer segment OS layer value was significantly lower in non-responders QLT091001 improved visual field and/or acuity in RP patients	High
13	Subretinal Visual Implant Alpha IMS—Clinical trial interim report	Katarina Stingl et al. (2015) [20]	International multicenter, single-arm, clinical trial	29 male and female patients with hereditary retinal degeneration (retinitis pigmentosa *n* = 25; cone–rod dystrophy *n* = 4). Patients had either light perception without projection (20 participants) or no light perception	Retina Implant Alpha IMS	Surgical implantation of microchip subretinally in one eye. Participants compared vision with implant’s power on or off. Follow-up for 1 year with visual function tests and monitoring	Primary: Improvements in daily activities and mobility. Secondary: Enhanced visual acuity and object recognition	72% showed better daily living and mobility 86% demonstrated improved visual acuity and recognition - Better detection and recognition of shapes and objects with the implant on - Improved gray level perception and light localization 86% experienced improved light perception and localization with the implant Few SAEs reported, mostly resolved successfully	High

## Data Availability

Data are available on reasonable request to the corresponding authors.

## Appendix A

Documentation on the literature search for: Retinitis pigmentosa and gene therapy, stem cell therapy, or optogenetics.

The following databases were searched:

**Database**	**Number of Retrieved Records**
MEDLINE (Ovid)	855
Embase (Ovid)	1339
Cochrane Systematic Reviews, Cochrane Register of Controlled Trials	85
Number of records before deduplication	2279
Number of records after deduplication	1612

All searches were performed on 21th of February 2024 by Hilde Iren Flaatten, medical librarian, University of Oslo Library of Medicine and Science.

Ovid MEDLINE(R) ALL (1946 to 20 February 2024)

1. retinitis pigmentosa/or alstrom syndrome/or bardet-biedl syndrome/or (retinitis pigmentosa or alstrom* or (bardet adj1 biedl) or biedl syndrome* or ((retina* pigment* or tapetoretina* or tapeto retina*) adj3 (dystroph* or degenerat*))).tw,kf.
2. genetic therapy/or rnai therapeutics/or targeted gene repair/or ((gene* adj3 (therap* or treatment*)) or (gene* adj3 repair*) or ((antisense or anti-sense) adj3 (therap* or treatment*)) or ((germ line or germline) adj3 (therap* or treatment*)) or ((oligonucleotide* or oligo-nucleotide*) adj3 (therap* or treatment*)) or (ribozyme* adj3 (therap* or treatment*))).tw,kf.
3. ((gene* or DNA or RNA) adj2 (immuni?ation* or vaccination*)).tw,kf.
4. ((gene* replacement* or mitochondrial replacement* or RNA interference* or RNAi or viral based or virus based) and (therap* or treatment*)).tw,kf.
5. Stem Cell Transplantation/or Induced Pluripotent Stem Cells/or stem cell*.tw,kf.
6. stem cells/or pluripotent stem cells/or embryonic stem cells/or human embryonic stem cells/or induced pluripotent stem cells/or (IPSC or IPS cell*).tw,kf.
7. Optogenetics/or (optogenetic* or opto-genetic*).tw,kf.
8. or/2–7
9. 1 and 8
10. exp models, animal/or ((Animal Experimentation/or exp Animals/) not Humans/) or (veterinar* or animal or animals or swine or rabbit or rabbits or rodent or rodents or rat or rats or mouse or mice or hamster or hamsters or pig or pigs or piglet or piglets or porcine or pigeon* or horse* or equine or cow or cows or bovine or goat or goats or sheep or lamb or lambs or monkey or monkeys or murine or ovine or dog or dogs or canine or cat or cats or feline or dolphin* or fish or zebrafish).ti.
11. 9 not 10
12. limit 11 to english language

Embase Classic+Embase (1947 to 20 February 2024)

1. retina pigment degeneration/or retinitis pigmentosa/or Alstrom syndrome/or Bardet Biedl syndrome/or (retinitis pigmentosa or alstrom* or (bardet adj1 biedl) or biedl syndrome* or ((retina* pigment* or tapetoretina* or tapeto retina*) adj3 (dystroph* or degenerat*))).tw,kf.
2. gene therapy/or antisense therapy/or cell based gene therapy/or exp gene replacement therapy/or exp genetic immunization/or germ line gene therapy/or nonviral gene therapy/or oligonucleotide therapy/or ribozyme therapy/or rnai therapeutics/or somatic gene therapy/or stem cell gene therapy/or viral gene therapy/
3. ((gene* adj3 (therap* or treatment*)) or (gene* adj3 repair*) or ((antisense or anti-sense) adj3 (therap* or treatment*)) or ((germ line or germline) adj3 (therap* or treatment*)) or ((oligonucleotide* or oligo-nucleotide*) adj3 (therap* or treatment*)) or (ribozyme* adj3 (therap* or treatment*))).tw,kf.
4. ((gene* or DNA or RNA) adj2 (immuni?ation* or vaccination*)).tw,kf.
5. ((gene* replacement* or mitochondrial replacement* or RNA interference* or RNAi or viral based or virus based) and (therap* or treatment*)).tw,kf.
6. stem cell transplantation/or induced pluripotent stem cell/or pluripotent stem cell/or embryonic stem cell/or human embryonic stem cell/or (stem cell* or IPSC or IPS cell*).tw,kf.
7. optogenetics/or (optogenetic* or opto-genetic*).tw,kf.
8. or/2–7
9. 1 and 8
10. exp animal model/or ((exp animal/or nonhuman/) not exp human/) or (veterinar* or animal or animals or swine or rabbit or rabbits or rodent or rodents or rat or rats or mouse or mice or hamster or hamsters or pig or pigs or piglet or piglets or porcine or pigeon* or horse* or equine or cow or cows or bovine or goat or goats or sheep or lamb or lambs or monkey or monkeys or murine or ovine or dog or dogs or canine or cat or cats or feline or dolphin* or zebrafish* or fish).ti.
11. 9 not 10
12. limit 11 to (conference abstracts or “preprints (unpublished, non-peer reviewed)”)
13. 11 not 12
14. limit 13 to english language

Cochrane

#1. MeSH descriptor: [Retinitis Pigmentosa] explode all trees
#2. (“retinitis pigmentosa” OR alstrom* OR (bardet NEAR/1 biedl) OR (biedl NEXT syndrome*) OR ((retina* pigment* OR tapetoretina* OR “tapeto retina” OR “tapeto retinal”) NEAR/3 (dystroph* OR degenerat*))):ti,ab,kw
#3. #1 OR #2
#4. MeSH descriptor: [Genetic Therapy] explode all trees
#5. ((gene* NEAR/3 (therap* OR treatment*)) OR (gene* NEAR/3 repair*) OR ((antisense OR “anti-sense”) NEAR/3 (therap* OR treatment*)) OR ((“germ line” OR germline) NEAR/3 (therap* OR treatment*)) OR ((oligonucleotide* OR (oligo NEXT nucleotide*)) NEAR/3 (therap* OR treatment*)) OR (ribozyme* NEAR/3 (therap* OR treatment*))):ti,ab,kw
#6. (((gene* OR DNA OR RNA) NEXT/2 (immuni?ation* OR vaccination*))):ti,ab,kw
#7. ((gene* NEXT replacement*) OR (mitochondrial NEXT replacement*) OR (RNA NEXT interference*) OR RNAi OR “viral based” OR “virus based”):ti,ab,kw
#8. (therap* OR treatment*)
#9. #7 AND #8
#10. MeSH descriptor: [Stem Cell Transplantation] this term only
#11. MeSH descriptor: [Induced Pluripotent Stem Cells] this term only
#12. MeSH descriptor: [Stem Cells] this term only
#13. MeSH descriptor: [Pluripotent Stem Cells] explode all trees
#14. ((IPSC OR IPS NEXT cell* OR stem NEXT cell*)):ti,ab,kw
#15. MeSH descriptor: [Optogenetics] this term only 2
#16. (optogenetic* OR opto NEXT genetic*):ti,ab,kw
#17. #4 OR #5 OR #6 OR #7 OR #10 OR #11 OR #12 OR #13 OR #14 OR #15 OR #16
#18. #3 AND #17

## Appendix B

	**Title**	**Include (*N*° of Eyes)**	**Exclude**	**Explanation for Exclusion**
1	Clinical trial of intravitreal injection of autologous bone marrow stem cells in patients with retinitis pigmentosa. ClinicalTrials.gov, 0(0). Retrieved from https://clinicaltrials.gov/show/NCT02280135 (2014), Access on 1 January 2020.		Excluded	Unpublished article
2	Zhao, T., Lie, H., Wang, F., Liu, Y., Meng, X., Yin, Z., & Li, S. (2021). Comparative study of a modified sub-Tenon’s capsule injection of triamcinolone acetonide and the intravenous infusion of umbilical cord mesenchymal stem cells in retinitis pigmentosa combined with macular edema. Frontiers in Pharmacology, 12. [9]	[1] 40 eyes (20 patients)		
3	Hoffman, D. R., Hughbanks-Wheaton, D. K., Spencer, R., Fish, G. E., Pearson, N. S., Wang, Y. Z., Klein, M., Takacs, A., Locke, K. G., & Birch, D. G. (2015). Docosahexaenoic acid slows visual field progression in X-linked retinitis pigmentosa: Ancillary outcomes of the DHAX trial. Investigative Ophthalmology & Visual Science, 56(11), 6646–6653. [16]	[2] 102 eyes (51 patients)		
4	Birch, D. G., Bernstein, P. S., Iannacone, A., Pennesi, M. E., Lam, B. L., Heckenlively, J., Csaky, K., Hartnett, M. E., Winthrop, K. L., Jayasundera, T., et al. (2018). Effect of oral valproic acid vs placebo for vision loss in patients with autosomal dominant retinitis pigmentosa: A randomized phase 2 multicenter placebo-controlled clinical trial. JAMA Ophthalmology, 136(8), 849–856. [18]	[3] 180 eyes (90 patiens)		
5	Russell, S., Bennett, J., Wellman, J. A., Chung, D. C., Yu, Z. F., Tillman, A., Wittes, J., Pappas, J., Elci, O., McCague, S., et al. (2017). Efficacy and safety of voretigene neparvovec (AAV2-hRPE65v2) in patients with RPE65-mediated inherited retinal dystrophy: A randomised, controlled, open-label, phase 3 trial. The Lancet, 390(10097), 849–860.		Excluded	Preliminary results of an included article/aim out of scope
6	Maguire, A. M., Russell, S., Wellman, J. A., Chung, D. C., Yu, Z. F., Tillman, A., Wittes, J., Pappas, J., Elci, O., Marshall, K. A., et al. (2019). Efficacy, safety, and durability of voretigene neparvovec-rzyl in RPE65 mutation-associated inherited retinal dystrophy: Results of phase 1 and 3 trials. Ophthalmology, 126(9), 1273–1285.		Excluded	Preliminary results of an included article/aim out of scope
7	Euctr, F. R. (2014). Study of SAR421869 in Patients With Retinitis Pigmentosa associated with Usher Syndrome Type 1B. [Journal Article]. https://trialsearch.who.int/Trial2.aspx?TrialID=EUCTR2012-002574-31-FR Access on 20 February 2024		Excluded	Unpublished article
8	Maguire, A. M., Russell, S., Chung, D. C., Yu, Z. F., Tillman, A., Drack, A. V., Simonelli, F., Leroy, B. P., Reape, K. Z., High, K. A., et al. (2021). Durability of voretigene neparvovec for biallelic RPE65-mediated inherited retinal disease: Phase 3 results at 3 and 4 years. Ophthalmology, 128(10), 1460–1468. [15]	[4] 58 eyes (29 patients)		
9	Cehajic-Kapetanovic, J., Xue, K., Martinez-Fernandez de la Camara, C., Nanda, A., Davies, A., Wood, L. J., Salvetti, A. P., Fischer, M. D., Aylward, J. W., Barnard, A. R., Jolly, J. K., Luo, E., Lujan, B. J., Ong, T., Girach, A., Black, G. C. M., Gregori, N. Z., Davis, J. L., Rosa, P. R., Lotery, A. J., Lam, B. L., Stanga, P. E., & MacLaren, R. E. (2020). Initial results from a first-in-human gene therapy trial on X-linked retinitis pigmentosa caused by mutations in RPGR. Nature Medicine, 26(3), 354–359. [14]	[5] (18 patients)		
10	Zhao, T., Liang, Q., Meng, X., Duan, P., Wang, F., Li, S., Liu, Y., & Yin, Z. Q. (2020). Intravenous Infusion of Umbilical Cord Mesenchymal Stem Cells Maintains and Partially Improves Visual Function in Patients with Advanced Retinitis Pigmentosa. Stem Cells & Development, 29(16), 1029–1037. [8]	[6] 64 eyes (32 patients)		
11	Tuekprakhon, A., Sangkitporn, S., Trinavarat, A., Pawestri, A. R., Vamvanij, V., Ruangchainikom, M., Luksanapruksa, P., Pongpaksupasin, P., Khorchai, A., Dambua, A., Boonchu, P., Yodtup, C., Uiprasertkul, M., Sangkitporn, S., & Atchaneeyasakul, L. O. (2021). Intravitreal autologous mesenchymal stem cell transplantation: a non-randomized phase I clinical trial in patients with retinitis pigmentosa. Stem Cell Research & Therapy, 12(1), 52. [12]	[7] 14 eyes (14 patients)		
12	Liao, D., Boyer, D. S., Kaiser, P., Kuppermann, B. D., Heier, J., Mehta, M., Joseph, A., Kammer, R., Mills, B., Yang, J., et al. (2021). Intravitreal injection of allogeneic human retinal progenitor cells (hRPC) for treatment of retinitis pigmentosa: a prospective randomized controlled phase 2b trial. Investigative Ophthalmology & Visual Science, 62(8).		Excluded	Meeting abstract/unpublished article
13	Ozmert, E., & Arslan, U. (2023). Management of retinitis pigmentosa via Wharton’s jelly-derived mesenchymal stem cells or combination with Magnovision: 3-year prospective results. Stem Cells Translational Medicine, 12(10), 631–650. [11]	[8] 130 eyes (80 patients)		
14	Limoli, P. G., Limoli, C. S. S., Morales, M. U., & Vingolo, E. M. (2020). Mesenchymal stem cell surgery, rescue and regeneration in retinitis pigmentosa: Clinical and rehabilitative prognostic aspects. Restorative Neurology & Neuroscience, 38(3), 223–237. [13]	[9] 34 eyes (25 patients)		
15	Hu, Y., Du, Y., Jin, Y., Feng, K., Chen, H., Han, L., Qu, H., & Ma, Z. (2023). A Novel Surgical Approach for Big Sheet Allogenic Retinal Pigment Epithelium-Bruch Membrane Complex Transplantation Into the Subretinal Space. Retina, 43(10), 1816–1819.		Excluded	Small case series
16	Hoffman, D. R., Locke, K. G., Wheaton, D. H., Fish, G. E., Spencer, R., & Birch, D. G. (2004). A randomized, placebo-controlled clinical trial of docosahexaenoic acid supplementation for X-linked retinitis pigmentosa. American Journal of Ophthalmology, 137(4), 704–718. [17]	[10] 88 eyes (44 patients)		
17	Scholl, H. P., Moore, A. T., Koenekoop, R. K., Wen, Y., Fishman, G. A., van den Born, L. I., … & Bowles, K. (2015). Safety and proof-of-concept study of oral QLT091001 in retinitis pigmentosa due to inherited deficiencies of retinal pigment epithelial 65 protein (RPE65) or lecithin: retinol acyltransferase (LRAT). PloS one, 10(12), e0143846. [19]	[11] 36 eyes (18 patients)		
18	Sobaci, G., Sevinc, K., Ovali, E., Ozmert, E., Ozdek, S., Yilmaz, G., … & Akkoyun, I. (2015). Submacular allogeneic ectomesenchymal stem cell transplantation in retinitis pigmentosa: one-year results. Investigative ophthalmology & visual science, 56(7), 2276.		Excluded	Small case series
19	Stingl, K., Bartz-Schmidt, K. U., Besch, D., Chee, C. K., Gekeler, F., Groppe, M., Jackson, T. L., MacLaren, R. E., Koitschev, A., & Kusnyerik, A. (2015). Subretinal Visual Implant Alpha IMS–Clinical trial interim report. Vision research, 111, 149–160. [20]	[12] 29 eyes (29 patients)		
20	Kahraman, N. S., & Oner, A. (2020). Umbilical cord derived mesenchymal stem cell implantation in retinitis pigmentosa: a 6-month follow-up results of a phase 3 trial. International Journal of Ophthalmology, 13(9), 1423–1429. [10]	[13] 124 eyes (82 patients)		
21	Russell, S., Bennett, J., Maguire, A. M., & High, K. A. (2018). Voretigene neparvovec-rzyl for the treatment of biallelic RPE65 mutation-associated retinal dystrophy. Expert Opinion on Orphan Drugs, 6(8), 457–464.		Excluded	Expert opinion
22	Euctr, G. B. (2016). A Clinical Trial of Retinal Gene Therapy for X-linked Retinitis Pigmentosa using AAV8. [Protocol]. Retrieved from https://trialsearch.who.int/Trial2.aspx?TrialID=EUCTR2016-003852-60-GB Accesed on 20 February 2024		Excluded	Unpublished article
23	A clinical trial to evaluate the effect of bone marrow-derived stem cells in diseases like dry age-related macular degeneration and retinitis pigmentosa. [Protocol]. Retrieved from https://trialsearch.who.int/Trial2.aspx?TrialID=CTRI/2010/091/000639 (2010) Accesed on 20 February 2024		Excluded	Unpublished article
24	Mohanty, S., Batabyal, S., Kim, S., Carlson, M., Ayyagari, A., Rittimann, J., Tchedre, K., & Chavala, S. H. (2022). Double-masked, Randomized, sham-controlled, Multicenter Phase 2b study of Multi-Characteristic Opsin enabled vision restoration in patients with advanced retinitis pigmentosa: design and Development of novel endpoints. Investigative ophthalmology & visual science, 63(7), 1722-F0040.		Excluded	Meeting abstract/unpublished article
25	Boyer, D. S., Bergstrom, L., Emanuelli, A., Gonzalez, V. H., Wykoff, C. C., Gupta, S., Liao, D. S., Zak, V., Chavala, S. H., Mohanty, S., et al. (2023). Efficacy and safety of MCO-010 optogenetic therapy for vision restoration in patients with severe vision loss due to retinitis pigmentosa: a phase 2b randomized, sham-controlled, multi-center, multi-dose, double-masked clinical trial (RESTORE). Investigative ophthalmology & visual science, 64(8), 5443.		Excluded	Meeting abstract/unpublished article
26	Nct. (2021). Efficacy and Safety of MCO-010 Optogenetic Therapy in Adults With Retinitis Pigmentosa. Journal of Clinical Trials, 0(0). Retrieved from https://clinicaltrials.gov/show/NCT04945772 Accesed on 20 February 2024		Excluded	Unpublished article
27	Euctr, D. K. (2021). Gene Therapy Trial for Patients with Retinitis Pigmentosa Due to a Gene Defect on Chromosome X. Journal of Clinical Trials, 0(0). Retrieved from https://trialsearch.who.int/Trial2.aspx?TrialID=EUCTR2020-002873-88-DK Accesed on 20 February 2024		Excluded	Unpublished article
28	Yusupov, A. Y. (1956). Implantation of catgut in the treatment of some eye diseases. Sborn, 0(0), 303-307. Retrieved from https://ezproxy.uio.no/login?url=http://ovidsp.ovid.com/ovidweb.cgi?T=JS&CSC=Y&NEWS=N&PAGE=fulltext&D=emcl1&AN=281398832 Accesed on 20 February 2024		Excluded	
29	Satarian, L., Nourinia, R., Safi, S., Kanavi, M. R., Jarughi, N., Daftarian, N., Arab, L., Aghdami, N., Ahmadieh, H., & Baharvand, H. (2017). Intravitreal Injection of Bone Marrow Mesenchymal Stem Cells in Patients with Advanced Retinitis Pigmentosa; a Safety Study. Journal of Ophthalmic & Vision Research, 12(1), 58–64.		Excluded	Aim out of scope
30	Liao, D., Gonzalez, V., Emanuelli, A., Gupta, S., Wykoff, C., Zak, V., Ayyagari, A., Bataybal, S., Chavala, S., Koester, J., et al. (2023). Optogenetic Therapy with MCO-010 for Vision Restoration in Patients with Severe Sight Loss Due to Retinitis Pigmentosa: the Phase 2b RESTORE Study. Molecular Therapy, 31(4), 399.		Excluded	Abstract/unpublished article
31	Euctr, N. O. (2021). A Phase 2/3 study to evaluate efficacy, safety, and tolerability of QR-421a in subjects with advanced vision loss. https://trialsearch.who.int/Trial2.aspx?TrialID=EUCTR2021-002729-74-NO Accesed on 20 February 2024		Excluded	Unpublished article
32	Euctr, N. O. (2021). A Phase 2/3 study to evaluate efficacy, safety, and tolerability of QR-421a in subjects with advanced vision loss. [Journal Article]. https://trialsearch.who.int/Trial2.aspx?TrialID=EUCTR2021-002729-74-NO Accesed on 20 February 2024		Excluded	Unpublished article
33	Nct. (2023). Role of UC-MSC and CM to Inhibit Vision Loss in Retinitis Pigmentosa Phase I/II. [Journal Article]. https://clinicaltrials.gov/show/NCT05909488 Accesed on 20 February 2024		Excluded	Unpublished article
34	Euctr, D. K. (2016). A study in subjects with rare inherited eye conditions caused by gene mutations to see if treatment with QLT091001 is safe and works to improve subjects’ vision.		Excluded	Unpublished article
35	Euctr, F. R. (2018). Study to evaluate QR-421a in subjects with retinitis pigmentosa (RP) due to mutations in exon 13 of the USH2A Gene		Excluded	Unpublished article
36	Euctr, G. B. (2008). An open-label dose escalation study of an adeno-associated virus vector (AAV2/2-hRPE65p-hRPE65) for gene therapy of severe early-onset retinal degeneration—RPE65 gene therapy.		Excluded	Unpublished article
37	Oner, A., Gonen, Z. B., Sinim, N., Cetin, M., & Ozkul, Y. (2016). Subretinal adipose tissue-derived mesenchymal stem cell implantation in advanced stage retinitis pigmentosa: a phase I clinical safety study. Stem Cell Research and Therapy, 7(1), 1–12.		Excluded	Small case series
